# Guy's Hospital Reports. Second Series

**Published:** 1844-10

**Authors:** 


### Art. V.

*Guy's Hospital Reports. Second Series.* Nos. I, II, and III. April, 1843, to April, 1844. *London,* 8vo.

After a prosperous course of seven years, the editors of these valuable Reports, having added to their own numbers, have determined upon extending the field of their labours also. In addition to the finished treatises, the works of individuals, which have chiefly occupied the former volumes, they now propose to illustrate the different classes of disease by the aid of a series of Reports, collected within the walls of the hospital, and furnished by the books of the Clinical Society, (attached to the institution;) and likewise, to apportion a part of each number to the consideration of anomalous cases from the same source. Original views may also be occasionally propounded concerning some of the sciences connected with medicine. We regard this plan as one that is calculated to increase the utility of the work in no small degree, if it be fairly and fully developed; and we therefore cheerfully lend ourselves to the task of analysing, after our accustomed manner, the portions which are now before us.

No. I. 1. The first paper which claims our notice is a *Case of suspected irritant poisoning,* by Mr. A. S. Taylor, which, like everything proceeding from the pen of that author, is full of interest. Three members of the family of a shepherd—the wife, son, and daughter, the two latter being young children—were taken ill on Sunday, after dining on some mutton from a sheep which had died of the '*staggers.*' They were better next day, and again partook, with the father, of the same food. In *less than three hours* the boy died; the mother and daughter were found, at the same time, in a state of insensibility; and the father felt, while at work, a sharp burning pain in his inside. No very accurate account of the symptoms could be procured; the mother foamed at the mouth, and was in a state of great nervous excitement; both the children, however, suffered from vomiting and purging, the boy especially. His motions were of a dark-green colour; the matters vomited were very copious, and striated with a yellow-coloured substance: they were, unfortunately, thrown away. On examination of the body after death, the lungs were found loaded with scarlet blood; the liver of a pink colour, and congested with liquid blood. On the posterior part of the stomach there were several inflamed and prominent rugæ, and other parts of the lining membrane presented traces of inflammation. The upper part of the small intestines was inflamed, and they contained a liquid mixed with blood. The peritoneum was highly inflamed; and there were two spots of well-defined inflammation on the posterior wall of the contracted bladder. The cavity of the abdomen contained about two ounces of bloody serum. The upper part of the larynx and lower part of the pharynx were inflamed. The vessels of the brain were congested. It is clear that many of these appearances were unconnected with the action of the deleterious agent, whatever that might be. No trace of poison could be detected in the contents of the stomach and viscera. Suspicion rested upon the father, but the evidence, both in a medical and moral point of view, was altogether inconclusive as to the administration, either wilfully or accidentally, of any mineral irritant poison; and we quite agree with Mr. Taylor in ascribing the death in this instance, and the illness in reference to the other parties, to the use of the diseased mutton. It is, however, a curious and not easily explained fact, that other families had partaken of the same meat without the production of any injurious effects. This may appear to militate against the view adopted, but it is not without parallel, for the author quotes an instance of poisoning by bacon, in which the same thing was observed. We believe this is the only example on record of poisoning by diseased or putrefied mutton, and it is also valuable as showing that death may occur in a much shorter time than has been hitherto imagined. We strongly recommend a careful perusal of the original paper, which contains, in addition to a minute analysis of the whole evidence bearing on this one particular point, a brief but interesting notice of all that is at present known in regard to animal poisoning.

2. *Observations on pelvic tumours obstructing parturition*, by S. C.W. Lever, M.D. This is a continuation of a paper in the seventh volume, and, like it, unsusceptible of condensation. It well merits perusal.

3. *Inquiry into the causes of death after injuries and surgical operations*, by Norman Chevers, M.D. A most important subject, especially when treated, as in this instance, with a view to the adoption of preventive means. There is great variety in the nature of these fatal actions, but they may be arranged in three general groups: viz. 1. The *primary*, occurring either during or very shortly after operations or injuries, such as collapse, hemorrhage, the admission of air into the veins, or the sudden occurrence of any internal lesion. 2. The *secondary*, including those which are developed within a few hours or days, such as tetanus, delirium tremens, irritative fever, secondary hemorrhage, sloughing, erysipelas, and acute inflammations of the various internal organs, of the veins and arteries. 3. The *remote*, which do not cause death until after an interval of some weeks or months, such as profuse suppuration, secondary fever, caries of injured bone, phthisis.

Dr. Chevers's remarks are chiefly confined to the inflammatory diseases included in the second class; and his object is to prove that, in a large proportion of cases, they may be traced to previously existing disease, or a predisposition to disease, in the kidneys, liver, and spleen, but especially in the former. His suspicions were first excited by observing that the morbid characters presented by the serous membranes and other structures, together with the appearances of the effused fluids, &c. in those who died of acute internal inflammatory attacks consequent upon operations or injuries, (especially where the primary wounds were at a distance from the parts afterwards involved,) almost invariably bore a precise resemblance to those which so characteristically distinguish the inflammatory affections of the same parts which are known to result from Bright's disease of the kidney. Subsequent inquiries demonstrated the correctness of this idea. Out of 134 cases in which the patients died of internal inflammations, there was also superadded marked disease of the kidneys, liver or spleen, or of all these organs combined, in 90; and from the character of the symptoms, &c. in many, of the whole number, in which the kidneys were not examined, Dr. Chevers feels confident that the proportion would have been still greater had the post-mortem scrutiny been more complete. In rather a large proportion of these cases the diseases above mentioned had evidently existed for a considerable period before the reception of the wounds or injuries which became the apparent primary causes of death: but in very many (and this was especially observable in the renal cases) the changes were manifestly of so recent a nature as to render it probable that almost immediately after the operations or accidents, either visceral disease had been excited from a latent to an active condition, or that a state of acute congestion had suddenly been established in organs which had hitherto been suffering merely from chronic disorder.

The practical inference to be derived from the above facts is sufficiently obvious, viz. that no operation should be performed upon any patient (excepting, of course, in those cases in which delay would be death), until the state of these important organs is satisfactorily ascertained, and any diseased action which may exist is, as far as possible, subdued. And though the observations here recorded are chiefly applicable to patients similarly situated with those who are the inmates of London Hospitals, yet we cannot but strongly recommend to *all* surgeons a most careful and anxious out-looking in the same direction.

4. *On the structure, functions, and diseases of the coronary arteries of the heart,* by Norman Chevers, M.D. There is considerable peculiarity in the structure of these vessels. The lining membrane is, of course, a prolongation of the internal tunic of the aorta, and like it has a minutely granular surface, when the secretion which maintains its polish is removed by a sponge or by drying. It cannot be separated from the sub-serous tissue. This tissue is a continuation of the very strong fibrous tunic of the aorta. At the commencement of the vessels the fibres form rather thin laminæ; but immediately upon reaching the interior of the arteries, they undergo a great increase of development, spreading out into several layers (three of which, at least, may be discerned upon very cursory examination), and, in short, forming the main tunics of the vessels. In extending along the tubes, these fibres do not take a perfectly longitudinal course; but those of the different layers, spreading out as they descend, cross the fibres of the laminæ above and below them at very acute angles. This arrangement is also found in the aorta, and greatly adds to the strength of the tubes.

The middle elastic coat is very thin, consisting, apparently, of a single layer of circular fibres, slightly interlaced. External to this, and easily separable from it, is the fourth tunic, strongly reticulated, and plentifully supplied with vessels, and surrounded, throughout the whole of its course, by the intricate network of nerves, from the cardiac plexus, which may almost be regarded as intended to form another covering for the defence of each vessel under distension. Dr. Chevers has observed a very similar arrangement of parts in the superficial veins of the extremities. The structure above described is admirably calculated to enable these vessels to sustain the impetus of the downward current of the blood, and to recover from the elongation which, at every injection, that current must produce in their canals; while at the same time it confers upon them sufficient lateral extensibility to enable them to bear those degrees of distension to which they must be frequently exposed, both when an increased quantity of blood is thrown into them under inordinate action of the heart, and when the passage of their contents into the coronary veins is obstructed by any cause. In reference to the *diseases* of these arteries, Dr. Chevers does not advance anything very new.

5. *On digestive solution of the œsophagus, with a case*, by Mr. T. Wilkinson King. A young man, who had been a hard drinker, had complained of epigastric pain, sickness, loss of appetite, and flatulence for some months. He was compelled to leave a public supper about nine o'clock on account of sickness; vomited slightly, and returned home, but could not walk without assistance. His wife then gave him some castor-oil. He was seen by a medical gentleman about three a.m. He complained of severe pain about the epigastrium and great difficulty of breathing; the abdominal muscles were very rigidly contracted; he was sitting up in bed, and leaning forward on his hands; countenance anxious and pulse soft. Remedies were of no avail. Emetics were administered, but no vomiting took place. The dyspnea increased; the face, throat, and chest were distended with emphysema, and he died at noon. On dividing the cartilages of the ribs on the left side, fluid of a dark and offensive character issued from the chest; the lung on the same side was collapsed, and castor-oil floated on the surface of the fluid in the pleural cavity. There were marks of inflammation on the outside of and within the pericardium. There was a large rent in the œsophagus, where it passes through the diaphragm, filled up with food; portions were also found at the posterior part of the chest. The left end of the stomach was softened by digestion.

We leave our readers to form their own opinion upon this case. To us it appears that the whole phenomena are most easily explained on the supposition of a rupture having taken place during life. Mr. King is of a different opinion, and rather imagines the death to have been caused by sudden inflammatory tumefaction of the larynx, of which we confess ourselves unable to detect any evidence. Nor do we see how the case is corroborative of his views of the different actions of the two ends of the stomach.

6. An interesting *case of glanders in the human subject*, by Dr. H. M. Hughes.

7. *Report of cases of hernia at Guy's Hospital, from Sept.* 1841 *to Dec.* 1842, by Mr. Alfred Poland. This is a lengthy paper, containing the record of forty-five cases; eighteen were the subjects of operation, and of these nine died. Was not too much time lost in the exhibition of remedies before operating in some of the cases? We cannot but think it is the duty of the surgeon to interfere within a very few hours, when the symptoms show no tendency to yield under the employment of the ordinary means. A reference to cases 8, 11, and 25 will, we think, bear us out in these remarks. Appended to the paper is an account of four anomalous cases, which are well deserving of a perusal. In one, fungoid disease of the testis and cord was mistaken for an omental hernia, and the fatal event, in all probability, much hastened by the vain efforts at reduction.

8. *Observations on patients whose urine was albuminous,* by Drs. Bright and Barlow. A valuable paper, well deserving an attentive perusal. Its great length, however, (upwards of 100 pages,) and the nature of its contents, which are for the most part the mere reports of cases, preclude the possibility of analysis within any reasonable limits. A number of the cases are so arranged as to exhibit the influence of particular remedies or courses of treatment; this plan we regard as a most excellent one, and shall be glad to see it more extensively followed. We have risen from the perusal, with a deepened conviction that a *remedy* for these diseases is yet among the things to be desired, and with our previously formed opinion, that *mercury* is decidedly unsafe, not a little strengthened.

How have the editors allowed so many typographical errors to escape their notice?

9. *Observations on the blood, with reference to its peculiar condition in the morbus Brightii,* by Dr. G. O. Rees. In a communication formerly noticed by us (Br. and For. Med. Rev., No. XIV, p. 133,) Dr. Rees explained his views of the structure of the red corpuscle, which he believes to consist of a closed sac containing a fluid, the sac being capable of exhibiting the phenomena of endosmosis and exosmosis. But he had not then determined whether the red colour existed in the sac or in the fluid. The following experiment shows that the latter is the case:

"The serum was decanted from a specimen of coagulated blood: the clot was next carefully washed in the serum, in order to get as many red corpuscles as possible into the liquor; it was then removed, and the serum set aside, to allow the red corpuscles to subside. Subsidence being complete, the supernatant fluid was decanted, and the deep red, thick mixture at bottom poured into distilled water, in order to burst the membranes of the corpuscles. This aqueous mixture was then well stirred and set aside; when, after a few hours had elapsed, I observed a white deposit at the bottom of the containing vessel, while the supernatant liquor remained clear, and of a fine red colour."

The white deposit, examined under the microscope, appeared to be made up of burst membranes of corpuscles, and of nuclei; and when the supernatant fluid was evaporated to dryness, and the dry mass incinerated, it was found to contain all the iron.

Dr. Rees believes that the iron, which gives colour to the fluid of the corpuscles, is communicated by what is called the *aqueous extractive* of the chyle, and which passes into the corpuscles by endosmosis; the specific gravity of the contents of the thoracic duct in the human subject being 1024, while that of the liquor sanguinis is about 1052, at least. If such be the case, it is quite clear that anything which alters the relative specific gravities of these two fluids must have an influence over the process above mentioned, and, consequently, affect the quantity of colouring matter in the blood. Thus, if the specific gravity of the liquor sanguinis be diminished, (as it undoubtedly is in the morbus Brightii,) there will be less endosmosis of the fluid matter of the chyle, and, by consequence, a deficiency of iron in the corpuscles. In this way Dr. Rees explains the diminution of red corpuscles in these renal diseases. The subject is one of much interest, and deserves further investigation.

10. *Report of cases of fever*, by Mr. J. H. Browne. From this paper we have derived no new information of any value; and we have again to complain of the carelessness with which the text is prepared. There are numerous grammatical blunders, (e. g. "the pulse *were*," in about half the number of cases,) and the style is altogether inelegant and devoid of precision.

No. II. 1. *Observations on pneumonia and its consequences*, by T. Addison, M.D. In this valuable and interesting communication the author confines his attention to certain points which appear to him to require or admit of further elucidation, passing over the general history of the disease. And, in the first place, he expresses doubts whether what is generally denominated *simple pneumonia* be in reality so uncomplicated an affection as is supposed. His own experience leads him to regard this form of the disease as a *broncho-pneumonia;* but he believes that a truly simple pneumonia is not very unfrequently met with in young patients, and in good constitutions, unattended by either cough, expectoration, or pain—at least, in such a degree as to attract particular attention. This important fact will be more fully noticed on a future occasion.

*Seat of pneumonia.* Our readers are doubtless aware that Dr. Addison dissents from the common opinion, that the inflammatory deposits are poured into an interstitial tissue, the very existence of which he has been unable to discover; and believes that the original and essential seat of pneumonia is in the air-cells. The question yet remains an open one, but we confess these views appear to us the most sound and satisfactory.

*Effects of pneumonia.* The first effect is probably a preternatural dryness of the air-cells, from an arrest of their natural secretions, which (also *probably*,) is characterized physically by an excited state of the respiratory act, and a murmur louder than natural. This stage is soon succeeded by an effusion of serum into the air-cells themselves—not into the intestinal tissue, as may be shown by subjecting a portion of lung thus affected to pressure, when the fluid will exude from the truncated bronchial tubes, and may be collected for examination. In this stage the cells are highly red and vascular, and already feel more substantial and dense than natural, though still crepitating and containing air. Sooner or later, however, the turgid parietes of the cells lose their natural cohesion, swell, and, encroaching on the cavities, cause the ab sorption of the serum previously effused; and thus occasion, first, a dryness and brittleness, and afterwards the complete consolidation which constitutes the red hepatization of authors, and which is accompanied by such a loss of cohesion as causes the lung readily to break down under pressure. As the inflammation proceeds, the parietes of the cells become more and more opaque and thickened; the minute blood-vessels are no longer visible; the tissues become exceedingly softened; and, with this extreme loss of cohesion and apparently diminished vascular turgescence, we have undeniable proof that the cells now admit of an albuminous matter being poured into their cavities. This constitutes the gray hepatization of authors. The degree of solidity of this effused matter will depend upon the strength of the constitution, and the sthenic form of the inflammation. These changes may occupy a large extent of the lung, or be limited to separate lobules, which latter most commonly occurs in cachectic subjects.

What is called *carnification* of the lung, and generally believed to be the result of pneumonic inflammation, modified by pleuritic effusion, Dr. Addison considers to be merely the effect of pressure sufficient to force out all the contained air; having ascertained that a portion of lung in this state, which instantly sunk in water, could be perfectly inflated, and then presented a natural and healthy appearance with the single exception of slight redness.

The above may be regarded as the more *immediate* changes produced by inflammation. The *permanent* effects, according to our author, present three varieties: 1, the uniform albuminous induration; 2, the granular induration; 3, the gray induration.

*a. The uniform albuminous induration* is the least frequent: it occurs occasionally as the result of acute pneumonia in good constitutions, the softened tissues appearing to become so blended with, or assimilated to, the permanent albuminous deposit, that the whole is converted ultimately into a uniform homogeneous, semi-transparent, or opaque and yellowish material, in which not the slightest trace of either the aërial cellular structure or of the common interlobular cellular membrane can be discovered. Most frequently a considerable portion of lung is affected in this way; but occasionally the change is limited to one, or a very few lobules.

*b. The granular induration* is caused by the effusion of a less organizable albumen. The interlobular cellular tissue often, but not necessarily, remains perfectly distinct; and a solid, pale, or yellowish and friable albuminous matter occupies the lobules, apparently without having assimilated the parietes of the cells; so that the lobules, with their still separate cells filled with this substance, have something of the configuration of a raspberry. To this change the term *inflammatory tubercle* is sometimes applied.

*c. The gray induration* is made up of a mixture of dull or yellowish-white and black matter, in variable proportions, the density increasing with the darkness of colour. This change differs from the uniform albuminous induration in the albuminization of the tissues, being much less complete, and from the granular matter in the deposit being of a more plastic or organizable kind. In short, the pneumonic inflammation may be said to have terminated in adhesion; the albuminous effusion having partially undergone organization and contraction, and thereby glueing together and hardening the aërial cellular tissue—the whole of the appearances being, consequently the result of permanent albuminous deposit, obstructed cells, and black pulmonary matter. The interlobular cellular membrane probably undergoes a similar change. All these morbid structures or deposits maintain their integrity by a very slender power; and, under the influence of slight disturbing or devitalizing causes, are liable to undergo softening, and thus give rise to abscesses of various characters and extent.

These morbid changes are frequently regarded as forms of tubercular infiltration, and many will therefore doubt their inflammatory origin; but that the above is the true view of their nature, Dr. Addison is convinced, for the following, among other reasons. We occasionally have direct proofs of a previous attack of acute inflammation within the chest. They are pretty uniformly accompanied by the clearest evidences of former inflammation, viz. old deposit on the pleura, immediately above the affected portion of lung; adhesions between the pleuræ in that situation; considerable puckering of the pleura, with accompanying contraction and diminution of size of the lung itself, sufficient, in some instances, to cause actual deformity of the thoracic cavity. They are more frequently than otherwise found in the middle and inferior lobes of the lung. And, lastly, there is often the total absence of a vestige of tubercle in other parts of the lung.

Into the general and important question of the inflammatory or non-inflammatory origin of tubercle, which is incidentally noticed by Dr. Addison, we have not space to enter; but we recommend to our readers the attentive consideration of his remarks, assuring them that in this, as in every other part of the paper, there is food for much and improving meditation.

The paper is illustrated by seven admirable coloured drawings.

2, (and 5,—No. III.) *Observations on lithotomy*, by Bransby B. Cooper, Esq. This paper, though somewhat carelessly written, is characterized by much sound sense and judicious surgery; and deserves a careful perusal.

3. *On the pathology of cells*, by Thomas Williams, M.B. This article is the commencement of a series of Reports of facts and observations obtained in the microscopical department of the hospital. It contains much that is interesting, though well known, and some few things that are new. On the present occasion we shall confine ourselves to the notice of certain points that have been ascertained regarding the *cells of the liver.*

The liver abounds in large, well-defined, spheroidal cells, having a distinctly biliary tinge, and a considerable quantity of granular amorphous material. They also, as was determined independently by Henle and Erasmus Wilson, present the peculiarity of having in their interior, as one of their normal constituents, adipose particles, which vary remarkably in number and size. In cases of *fatty degeneration* Mr. Bowman first pointed out that the morbid change was caused by an unnatural accumulation of these particles in the interior of the cells; and the researches reported by our author have brought to light the following additional circumstances.

No fat-globules have ever yet been detected in the interior of the

*nucleus;* they are invariably situated between it and the outer or cell-membrane. As they accumulate, the nucleus completely disappears; the granular or primitive molecules undergo absorption; and with their removal the *yellow* tinge, or *bile-colour*, of the cell also disappears.

A patient died in the hospital from malignant disease of the duodenal extremity of the pancreas, which had so pressed upon the common excretory duct, that the passage of the bile into the duodenum had become almost impossible. The liver was considerably enlarged; it had become soft and flabby, and not capable of being easily broken down by pressure. On repeated examination of different portions of the organ with the microscope, scarcely a single nucleated glandular cell, in a perfect state, could be found. In each portion nothing more than minute, *free*, fatty particles, and equally free, floating, amorphous, granular matter could be discovered: it was very seldom that a *whole* nucleated cell could be seen. It would seem, therefore, that the pressure of the constantly accumulating bile had caused rupture of the cell-membranes.

In *fever* the adipose globules almost completely disappear from the cells of the liver.

In cases of *granular liver* the cells of the affected parts were found transparent and *colourless*, with few granules, but still containing adipose particles.

We shall be glad to meet with Dr. Williams again, but will venture to give him a gentle hint, (and it is meant in kindness,) that the value of his communications would be considerably enhanced by the adoption of a more *simple* style of writing; grandiloquence is altogether out of place in a professedly scientific paper.

4. Mr. Williams relates a successful case of *extirpation of the superior maxillary bone.*

5. *On a feculent discharge at the umbilicus, from communication with the diverticulum ilei*, by T. Wilkinson King, Esq. The author is of opinion that the diverticulum ilei is the remains of the canal which, in the earlier periods of fetal life, forms a communication between the umbilical vesicle and the parts within the abdomen; and which is described by Michel and others as the starting-point for the development of the bowels, both upwards and downwards. When the opening at the umbilicus remains patent, the object to be aimed at in treatment is to secure its closure, and to avoid cicatrization between the skin and mucous tube. This was effected, in the case before us, by making an incision of an ovoid shape round the orifice, and bringing the cut parts into apposition; caustic having been previously tried with only partial success.

6. *On the extirpation of ovarian cysts*, by Mr. C. Aston Key. Mr. Key advocates the long incision; but the only case in which he has operated, and the details of which are given in this place, was unsuccessful.

7. *Case of extra-uterine fetation*, by Mr. B. C. B. Rose, with a description of the parts, by Dr. Oldham.

8. *Cases of puerperal convulsions*, by Dr. S. C. W. Lever. The most interesting circumstance noted in this paper is the fact that, in nine out of ten cases, in which the condition of the urine was examined, it was found to contain *albumen* in variable proportions. The water, in every instance, was carefully drawn off by the catheter, so as to avoid mixture with the vaginal discharges. Dr. Lever has also examined the urine, obtained in the same way, from upwards of fifty women during labour, and with the following result: *in* NO *cases was albumen detected, except in those in which there were convulsions, or in which symptoms presented themselves which are readily recognized as the precursors of puerperal fits.*

Cases of convulsions thus complicated are divisible into two forms. In the one the urine is *albuminous during pregnancy;* and there are external evidences, as shown in œdema of the face, eyelids, hands, &c. In these cases the convulsions are more violent, and last for a longer time after delivery than in the other variety. The urine also retains its albuminous properties longer. In the second form the urine only becomes albuminous *during the labour;* the abnormal ingredient is less in quantity, and speedily disappears, and the fits seldom recur after delivery. Dr. Lever believes that the *causa mali* is the pressure of the gravid uterus upon the emulgent veins. The treatment advocated is, active depletion, when the patient will bear it, the free use of tartar-emetic, purgation, and as speedy a delivery as can be safely effected. He only gives mercury as a purgative; and remarks that its exhibition, even in this way, is not unattended with risk.

9, (and 6,—No. III.) *Report of cases of stricture of the urethra, retention, and extravasation,* treated from October, 1841, to October, 1842. We recommend this instructive record to all our surgical readers, and more especially to such of them as may be inclined to advocate the *heroic* practice of catheterism. The appended plate is full of solemn warning.

No. III. 1. *Cases of pelvic inflammation, with abscess, occurring after delivery,* by Dr. Lever. We have only room for a short abstract of the remarks made by the author on this affection, which has not received as much notice from obstetric writers as its importance demands. From the cases narrated, it would seem that the *seat* of the disease is the uterine appendages and the cellular tissue of the pelvis, but in which of these it originates does not appear so clearly. It may follow an attack of acute inflammation; or remain as the sequent of puerperal fever; or be caused by cold, falls, blows, &c. In only two of Dr. Lever's cases were the labours unnatural. The *symptoms* may commence a day or two after delivery, or a much longer period; some weeks even may intervene. The attack is generally preceded by rigors, or a sensation of coldness over the surface, followed by heat of skin, quickened circulation, and pain in the region of the pelvis. The febrile paroxysms are sometimes irregularly remittent. As the disease advances the pelvic pain increases; there is usually some degree of stiffness in the side affected, and, not unfrequently, pain in the course of the vessels of the thigh and leg: this may proceed to the development of phlegmasia dolens. The pulse is seldom below 100 or 110; the tongue loaded; urine scanty and high coloured, and passed at short intervals; bowels constipated at one time, and relaxed, with tenesmus, at another. In some cases a swelling is readily seen at the seat of the affection; in others there is merely an appearance of fulness on one or both sides. The parts are very sensitive, and feel hard. This hardness may extend as high as the umbilicus, or be confined to the pelvis. On examining *per vaginam*, in some cases, nothing abnormal may be detected, beyond increased size of the uterus. But in others the upper part and side of the vagina will be found hard, tender, firm, and inelastic; and by pressing upon the swelling felt through the abdominal parietes with one hand, and keeping the forefinger of the other in the canal, it will be found that the hardness and swelling in both situations arise from the same cause. Frequently there is some lateral displacement of the uterus. In some cases the swelling may be felt, *per rectum*, encroaching upon the bowels. The disease may terminate by *resolution* or, what is unfortunately more common, by *suppuration;* in which case the abscesses have been found to evacuate themselves, 1, externally; 2, into the cavity of the peritoneum; 3, into the vagina—the most frequent event; 4, into the uterus; 5, into the bladder; 6, into the intestines; 7, into the surrounding cellular tissue. The *sequelæ* may be, immobility of the uterus, leading to abortions in any subsequent gestations; an impervious condition of the Fallopian tube; and, probably, ovarian disease.

In the *treatment* heroic measures are neither required nor advisable, as the patients are generally those whose constitutional powers are much depressed. Blood should be taken by leeches as often as may be found necessary, encouraging the bleeding by warm sedative cataplasms, and vaginal injections of a similar nature, as the decoct. conii. Mild mercurials should be cautiously exhibited, to affect the system slightly; and the secretions carefully attended to, The distressing tenesmus may be relieved by the enema amyli, with syrup. papaveris, or tinct. opii, or by opiate suppositories. When it appears evident that suppuration is taking place, that process should be hastened by medicated poultices and fomentations; and, as soon as practicable, the abscess should be opened. M. Martin recommends the application of caustic potass to the abdominal parietes, for the double purpose of having an external opening, and securing previous adhesion of the containing sac to the abdominal walls. At the same time, the patient's strength must be supported by generous diet. If nursing appears to do injury, the child should be at once removed.

2. *Cases of poisoning*, by Mr. A. S. Taylor. The first case is that of a man who voluntarily swallowed about two drachms of corrosive sublimate. He presented the usual symptoms, but with some peculiarities. Thus, salivation began to make its appearance in about four hours after the poison had been taken, which is an unusually early period. He had also complete suppression of urine during the whole period that he survived—upwards of three days. The post-mortem appearances were also in some degree remarkable. Though life was so long protracted, there were no marks of corrosion, ulceration, or any tendency to perforation of the stomach, which was simply inflamed, very much as it appears in cases of arsenical poisoning. The slate-gray colour which the mucous membrane sometimes presents was entirely wanting. The duodenum and jejunum had entirely escaped the action of the poison, the chief effect produced on the small intestines being seen on the lower part of the ileum. The patient was subjected to most active antidotal treatment, but, as the above shows, without effect. *No trace of the poison could be detected upon the most careful chemical analysis.* The importance of this fact, in its bearing upon legal medicine, is self-apparent. It may be well to state, that, according to Mr. Taylor's experience, the most delicate test of corrosive sublimate is a coil of gold and zinc suspended for some hours in the suspected liquid, previously slightly acidulated with muriatic acid. If the poison be present, the gold will become tarnished, and from it metallic mercury may be obtained by heating in a reduction-tube. In this way Mr. Taylor has detected the $\frac{1}{144}$ of a grain, dissolved in one fluid drachm; that is, in more than eight thousand times its weight of water. About twenty-four hours is necessary for the production of the change when the quantity is so small.

The second case is an exceedingly instructive example of the dangerous effects of opium in young subjects. Death in this instance was caused by the administration of paregoric elixir to a girl aged five and half years, who was labouring under slight indisposition from a cough. We are unable to condense the reporter's valuable remarks, but would strongly recommend them to our readers.

3. *On paracentesis thoracis*, by Dr. Hughes and Mr. Cock. In the experience of these gentlemen, this operation has proved much more successful than other observers have found it. A table at the end of the paper gives an abstract of twenty cases of empyema, chronic pleuritic effusion, pneumothorax, &c. occurring in the practice of several gentlemen, and of which some records have been preserved. From this it appears that in ten the operation was followed immediately by great relief; in six by relief; in three there were no immediate effects; and in one the patient appeared in danger of suffocation, but ultimately recovered perfectly. Of the whole number of cases, seven were completely cured, and three recovered partially. Nine of the patients died; but of these six were phthisical, and no permanent good was anticipated from the operation; in one the fluid was not reached by the trochar; another was already sinking, under the complication of pneumonia and pericarditis, when the chest was opened; and in the remaining case, in which there was hydrothorax of the other side, death took place, rather suddenly, six days after the operation. One case still remained under treatment when the table was drawn up. In no instance could the fatal event be said to have been hastened by the operation.

We have not space to enter into the diagnosis of fluid in the cavity of the pleura, which is fully discussed in the paper before us, but must say a few words upon the manner in which they recommend the operation to be performed, and which, in some measure, differs from the more common method. The best spot for the puncture is below the angle of the scapula, between either the seventh and eighth or the eighth and ninth ribs, and at a point distant from one to three inches from the angle of the bone. In this situation there is least danger of wounding the diaphragm, and here we are most likely to procure a free discharge of the effusion. But, in many cases, it is advisable to make an exploratory attempt before proceeding to open the chest; and for this purpose a contrivance invented by Dr. Babington is admirably adapted. It consists of a needle contained in the smallest-sized canula; this is passed between the ribs into the suspected spot; the needle is then withdrawn, and the escape of fluid from the tube at once indicates the existence and the nature of the abnormal secretion. This instrument appears to us to possess many advantages over the ordinary grooved needle. Having thus determined upon the propriety of further proceedings, the trochar and canula are to be introduced in the same spot. Mr. Cock uses a circular instrument, which is about $\frac{1}{12}$ inch in diameter, and about two inches in length, exclusive of the handle. This is less than the usual size, but it has the advantage of being more easily introduced, of giving very slight pain, of ensuring a slow and gradual evacuation of the fluid, and of enabling us to avoid the admission of air; all which, but especially the last two, are matters of great importance. The patient should sit *across* the bed, so that his body may be readily lowered and supported over its edge. The introduction of the trochar will be much facilitated by making a small puncture in the skin with a narrow-bladed lancet. When the trochar is first withdrawn the fluid will issue in a steady and equable stream, but after a time the flow will be checked at each inspiration, and then the body of the patient should be gently lowered into a horizontal posture, and turned slightly to the affected side, so as to bring the cavity directly over the opening. Should the stream again begin to flag, steady pressure may be made on the lower part of the chest, by an assistant grasping it on either side with the hand; but as soon as these means fail in procuring a continuous flow the canula should be immediately withdrawn, while the chest is still compressed, that so the entrance of air may be prevented. The occurrence of this accident is known at once by the production of a peculiar sucking noise; and at whatever stage that sound may be heard, it should be the signal for the prompt removal of the instrument. The wound requires nothing but the application of a small dossil of lint and a strip of plaster.

4. *On polypus uteri, and its coexistence with pregnancy,* by Dr. Oldham. Uterine polypi are of various kinds. The most common is the *fibrous.* Such tumours may originate in any portion of the uterus; but they usually spring from some part of the body, its sides, or fundus. They consist of a fibrous growth, with more or less of uterine structure, (the latter being most abundant in the stalk,) covered by the mucous membrane of the womb. The anatomical elements are a clear, unstriped fibre, closely packed, interspersed in some instances with crystalline calcareous grains, and minutely divided arteries. The veins, though closely collected around the growth, do not appear to enter it. When these polypi descend, the walls of the uterus, to which they are attached, sometimes become inverted, and the depressed portion may be mistaken for the pedicle and tied, a circumstance full of danger. The uterine sound, as recommended by Dr. Simpson, of Edinburgh, will afford valuable aid in the diagnosis of such cases. Dr. Oldham relates a very remarkable case, which occurred lately to Dr. Rigby, in which the entire uterus was thus inverted, and removed by ligature, *without injury to the patient.*

Another variety of polypus is that which is called *spongy, cellular,* or *fibro-cellular.* The latter term correctly expresses its appearance when bisected; but, according to our author, gives an erroneous idea of its true nature; the void spaces or cells being, in most instances, truncated and divided veins, For in this species, the veins are not only collected in large trunks around the growth, but penetrate the centre, and are distributed through it in large channels, having a free communication with each other. In this situation their coats are supported by an unstriped fibre, closely resembling, if not identical with, the muscular tissue of the uterus.

Dr. Lee has described a form of polypus which is strictly *cellular.* Dr. Oldham has met with the same, and believes that it is formed by an expansion of the uterine glands.

The crypts of the cervix uteri furnish, in their enlargement, another variety; and the author has met with two specimens, situated in the same locality, which he designates the *channeled polypus of the cervix,* from the fact that its interior is made up of numerous large channels, with occasional communications between them, and opening, by large orifices, on the free surface of the growth. The channels are filled with transparent, sticky mucus.

Dr. Oldham believes that the hemorrhages in polypi are from the tumours themselves, and principally from the veins on their surface or pedicle; that sometimes the veins are lacerated, and that at others they open, under the accumulation of blood in them; as they do during menstruation.

The remainder of the paper is occupied by a narration of cases illustrating the coexistence of polypus with pregnancy, in reference to which we find the following summary:

"1. That various sized polypi, and some very large, may be developed in the uterus during pregnancy, without at all interfering with gestation, or in any way impeding parturition.

"2. That they are to be suspected when the uterus remains larger than usual after parturition, although hard and contracted, and when ineffective uterine efforts continue, attended by hemorrhage; and that they are liable to be mistaken for a secondary fœtus.

"3. That the distinction between a polypus and a second fœtus ought to be carefully made out before any attempts to deliver are begun; as, by confounding them, a polypus has been perforated, and the uterus rent and injured both by the hand and the forceps.

"4. That the hemorrhage they occasion is sometimes slight; at others, sudden and overwhelming: sometimes it occurs immediately after the birth of the child, or placenta; at others, it is delayed for a fortnight or three weeks; but it is frequently a harassing daily loss, which defies ordinary remedies to arrest it, and usually occurs in gushes.

"5. That the uterus may propel the polypus before the child's head; or, after delivery, it may close over the growth, and remain quiescent; or paroxysms of uterine pain may continue, with bleeding, or constant unremitting action of the womb, causing severe distress, exhaustion, and death.

"6. That the uterine action may occasion, in some instances, the separation of the growth; in others, the partial or complete inversion of the womb. If the polypi be attached to the cervix, or immediately above it, it may propel it into the vagina, separating perhaps some cellular adhesions; or, lastly, that its constant forcings with the weight of the polypus, may prolapse the uterus, and force the polypus beyond the external parts.

"7. That the treatment must depend on the prominent symptoms, and on the practicability of applying mechanical means: that if no hemorrhage be present, or it be but very slight, every endeavour, by opiates and rest, should be made to quiet the womb, until the shock of parturition, the uterine circulation, and that of the polypus, have diminished; that, if the hemorrhage be violent or continuous, and intractable, or if the incessant contractions of the womb threaten the life of the patient, the polypus is to be removed.

"8. That a polypus may be encircled by a ligature, or after that, be excised, or primarily twisted off, both during, immediately after, or at any time succeeding parturition, without the necessary production of bad symptoms; but that the ligature alone, or ligature and excision, are the preferable plans of operating.

"9. That a polypus thus removed is not regenerated; nor does it necessarily cause sterility, or occasion untoward symptoms in succeeding labours."

7. *Report of cases illustrating diseases of the brain and nervous system.* This paper is interesting, but unfitted for analysis.

---

